# Influence of a Three-Month Mixed Reality Training on Gait Speed and Cognitive Functions in Adults with Intellectual Disability: A Pilot Study

**DOI:** 10.3390/s24061821

**Published:** 2024-03-12

**Authors:** Alexis Laly, Elisabeth Rosnet, Nicolas Houel

**Affiliations:** Université de Reims Champagne-Ardenne, PSMS, 51100 Reims, France; elisabeth.rosnet@univ-reims.fr (E.R.); nicolas.houel@univ-reims.fr (N.H.)

**Keywords:** intellectual disability, virtual reality, mixed reality, gait, cognitive assessment, perceptual-motor task

## Abstract

People with intellectual disability (ID) are often subject to motor impairments such as altered gait. As gait is a task involving motor and perceptive dimensions, perceptual-motor training is an efficient rehabilitation approach to reduce the risk of falls which grows with age. Virtual, augmented, and mixed reality are recent tools which enable interaction with 3D elements at different levels of immersion and interaction. In view of the countless possibilities that this opens, their use for therapeutic purposes is constantly increasing. Therefore, the aim of this study was to investigate the influence a mixed reality activity could have on motor and cognitive abilities in eighteen adults with intellectual disability. For three months, once a week, they had around 20 min to pop virtual balloons with a finger using a Microsoft HoloLens2^®^ head-mounted mixed-reality device. Motor skills were assessed through gait analysis and cognitive abilities were measured with the Montréal Cognitive Assessment. Both walking speed and cognitive score increased after training. In conclusion, this study demonstrates that mixed reality holds potential to get used for therapeutic purposes in adults with ID.

## 1. Introduction

Intellectual disability (ID) is a neurodevelopmental disorder that begins in childhood and is characterized by intellectual difficulties as well as difficulties in conceptual, social, and practical areas of living [1] (American Psychiatric Association, 2013). Although it is not mentioned in the definition provided by the American Psychiatric Association, people with ID are frequently subject to delayed motor development [2], often have an impaired gait pattern [3,4], and lower perceptual-motor coordination [5], compared to healthy subjects. It seems important to focus on gross motor skills in intellectual disability since falls occur twice as often and sooner in the life of adults with ID than in healthy pairs [6].

It is not surprising to witness gait disturbance in ID since motor and cognitive systems are closely linked, especially in their development [7]. Furthermore, Azadian et al. [8] showed that training working memory could improve the gait pattern and walking speed of older adults. Conversely, Hillman et al. (2008) [9] suggest that physical activity can have a positive effect on multiple aspects of cognition. Thus, gait can appear as a complex cognitive–motor activity [10] when performed in specific environments. Cognitive–motor interactions during gait can be explained by a sharing of attentional resources [11] or by the existence of neural structures that are common to the use of cognitive and motor functions [12].

In the light of these cognitive–motor interactions and thanks to recent technological developments, new therapeutic approaches are emerging. Among them, the use of extended reality technologies (virtual reality, VR; augmented reality, AR; mixed reality, MR) is growing [13,14,15,16]. Klinger et al. define virtual reality (VR) as a scientific and technical field allowing an individual to interact in real time with 3D entities by means of behavioral interfaces, in an artificial world in which they are to some extent immersed [17]. The use of VR devices can promote motor learning and balance improvement in healthy subjects [18] but also in pathological populations (e.g., cerebral palsy and Down syndrome, [19]). Lotan and Weiss (2020) also showed balance improvement in adults with intellectual disability after training in a virtual environment [20].

**Figure 1:**
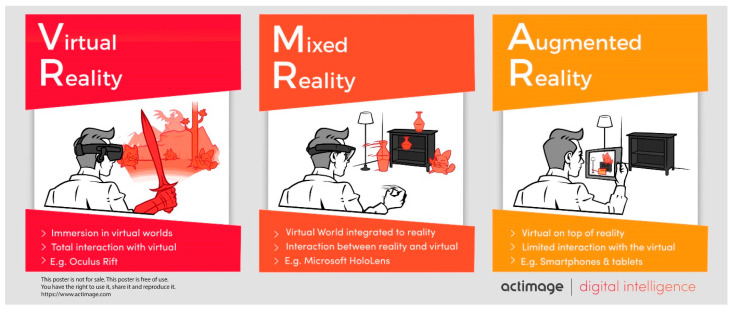
Extended reality technologies: VR; MR; AR (https://actimage.com (accessed on 9 February 2023, free of use)).

Nevertheless, one of the main limits to the use of VR, especially with head-mounted devices, is cybersickness [21]. Cybersickness is defined as the cluster of symptoms that a user experiences during or after exposure to an immersive environment [21]. Considered as a visual-induced motion sickness, its symptoms are like usual motion sickness (i.e., disorientation, nausea, oculomotor difficulties [22]). The occurrence of these symptoms can be significantly reduced with mixed reality devices (i.e., augmented reality head-mounted devices) [23]. As VR, mixed reality (MR) enables the user to interact with virtual holographic 3D objects. However, in MR they are overlaid on the real physical environment by means of an adapted head-mounted device (i.e., HoloLens^®^, Microsoft, One^®^, Magic Leap) (Figure 1). Hence, in a MR environment such as Microsoft HoloLens^®^ provides, the user perceives more visual information from the real physical environment than in VR, which reduces sensory conflict and therefore cybersickness [24]. Thus, MR seems suitable for rehabilitation purposes.

Using MR can improve balance in healthy subjects [25] but its influence on motor skills in people with ID has not been studied yet, except for the study by Laly et al. [26]. These first results suggest a positive effect of MR training on different gait parameters of subjects with ID (e.g., increased walking speed), despite a low training load (15–25 min/week) [26]. MR may have a strong therapeutic interest for this population because it can provide cognitive, perceptive, and motor stimulation for the user. Perceptual-motor training can improve motor skills in either healthy or pathological populations [27,28,29,30] and motor training can improve cognitive abilities [9]. Furthermore, extended reality technologies such as VR or MR allow for immersive and playful activities. As a result, subjects enjoy participating and their involvement is therefore enhanced [14,31].

Hence, the aim of this study was to investigate the effect of MR training on gait spatiotemporal parameters (STPs) and cognitive abilities of adults with ID. The primary hypothesis was that STPs are improved after training, especially walking speed, supporting the results of Laly et al. [26]. The secondary hypothesis was that the cognitive abilities may also increase.

## 2. Materials and Methods

### 2.1. Participants

The participants were 18 adults (6 women, 12 men) with mild to moderate intellectual disability. They were recruited in a home for disabled adults and in a “day activity center” for adults with disabilities, which were both parts of the same care organization. The sample characteristics are presented in Table 1. All subjects could understand simple indications; had a normal or corrected to normal view; accepted wearing the MR headset and could walk alone and without a walking aid tool. People with epilepsy or hallucinatory disorders were not included in the study to avoid seizures during or after the MR activity sessions. Down syndrome was a non-inclusion criterion to reduce the heterogeneity of the sample and not to include subjects whose motor impairment was linked to their genetics, as Down Syndrome is a chromosomal anomaly.

### 2.2. Experimental Procedure

#### 2.2.1. Study Design

**Figure 2:**
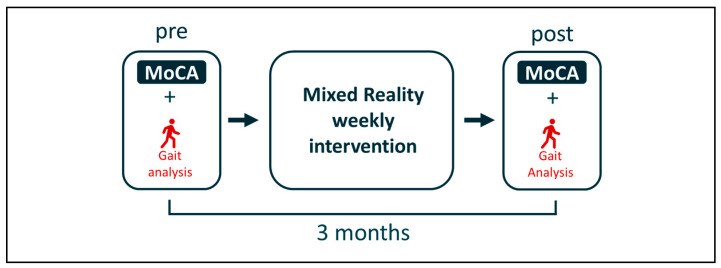
Study design.

After being included, subjects participated in a protocol for 3 months. The study design was made up as follows: an initial measurement session during which the subjects’ cognitive functions were assessed (see Section 2.2.2) before their gait was analyzed (see Section 2.2.3); a weekly mixed reality intervention; and a final measurement session identical to the initial session (Figure 2). There was one week between the first measurement and the beginning of the intervention, and one week between the end of the intervention and the final measurement. Measurement conditions (material; indications; investigator; place; time; etc.) were maintained between the pre- and post-intervention measurement times. The intervention and measurements are detailed in the following parts.

#### 2.2.2. Cognitive Assessment

The subjects’ cognitive abilities were assessed before (pre) and after (post) training with the French paper version of the MoCA (Montréal Cognitive Assessment [32]). The MoCA was originally designed for detecting mild cognitive impairments in older people [32]. However, it is also used as a general cognitive assessment tool [33,34,35] since it enables a quick overall cognitive assessment through a set of exercises involving cognitive functions (i.e., visuospatial; executive functions; naming; memory; attention; language; abstraction; time; and space orientation). While taking the test, every subject sat at a table in front of the investigator and in the presence of a psychological aid worker to form the structure, who was not allowed to intervene nor to help the subject. The MoCA test provides a score out of 30 points and in its casual use, a score below 26 out of 30 screens mild cognitive impairment [32].

#### 2.2.3. Gait Analysis

Gait spatiotemporal parameters (STPs) were assessed before (pre) and after (post) training using a Zeno™ walkway analysis system (ProtoKinetics LLC, Havertown, PA, USA) [36], combined with PKMAS™ 509c2 software (ProtoKinetics LLC, Havertown, PA, USA) [37]. Subjects were asked to walk shoeless at a comfortable and spontaneous pace, from side to side of the room along a straight way (i.e., approximately 12 m). As the active length of the mat was 5.48 m long, the acceleration and deceleration phases at the beginning and end of each passage were not measured to assess only stable speed gait [36,38]. Each subject performed six to eight round trips. If a participant stopped on the way, the entire one-way passage was removed from analysis to exclude acceleration and deceleration phases.

Gait spatiotemporal parameters (i.e., walking speed; cadence; walk ratio; step and stride lengths; stride width; step time; stride time; stance phase; single support time; double support time; initial double support time; swing phase; and feet angle) were automatically calculated by PKMAS^TM^ (ProtoKinetics LLC, Havertown, PA, USA) [37]. All STP values were obtained by calculating the left–right mean, except for “feet angle asymmetry” which is the left–right angle difference absolute value.

#### 2.2.4. Mixed Reality Intervention

**Figure 3:**
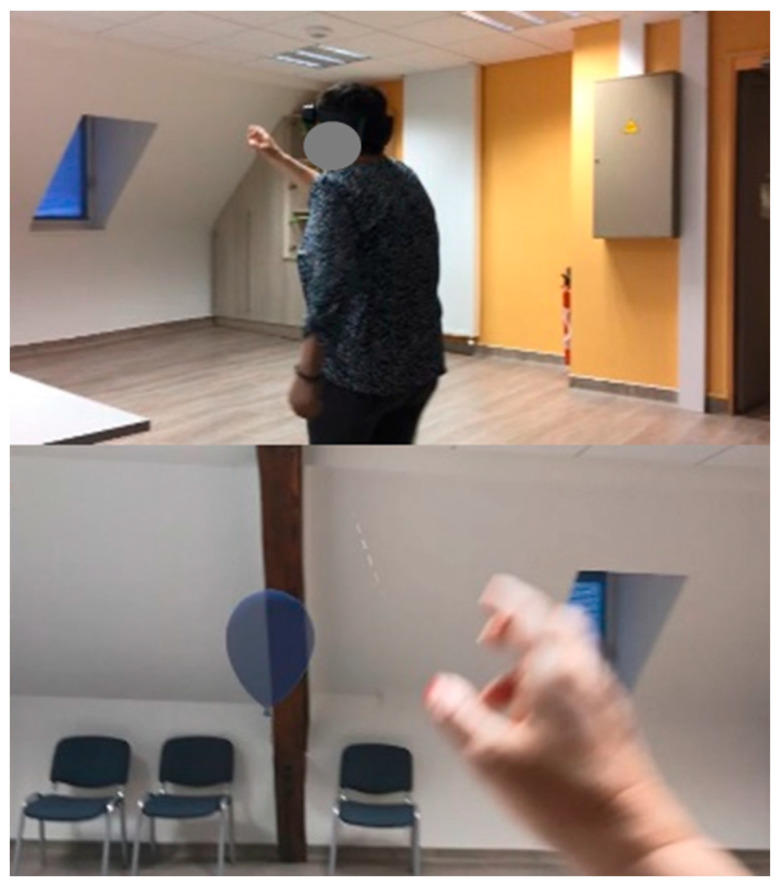
At the top: a subject playing the mixed reality game PopBalloons (Actimage, France) using the HoloLens2^®^ headset (Microsoft, USA). At the bottom: what a subject sees in the glasses at the same time—a virtual balloon in the real physical environment, which they are about to pierce with their finger. The balloon is a real-size 3D model appearing in the game area with a random position. In the picture, the balloon is approximately 1.5 m from the subject.

The weekly mixed reality activity consisted of using the application PopBalloons™ (Actimage, Paris, France) on a HoloLens2^®^ MR headset (Microsoft, Redmond, WA, USA). This is a game in which subjects see their real physical environment, which is augmented by virtual holographic balloons (Figure 3). Balloons appear randomly in a 3 m × 4 m area and the user has to seek them out, detect them, and pierce them with a finger. Only one balloon is displayed at a time and a new one appears as soon as the previous one is pierced. A level ends when five balloons have been touched. The weekly training lasted 30 min for each subject, but the effective time of activity was only around 20 min, considering breaks, headset setting on the subject’s head and level transitions. Subjects were asked to complete each level as fast as possible.

#### 2.2.5. Statistical Analysis

Data were processed in Matlab. For each parameter (i.e., STP and MoCA score), the normality of the distribution within the sample was tested with a Shapiro–Wilk test for pre- and post-intervention data. If the normal distribution was respected in both conditions, the significance of the pre–post difference was then tested with a Student’s *t*-test for paired series. If not, a Wilcoxon signed rank test, the non-parametric equivalent, was used. Hence, a Student’s *t*-test for paired series was used to compare the pre and post values of walking speed; step length; stride lengths; step time; stride time; stance phase; single support time; double support time; initial double support time; swing phase; and feet angle. The Wilcoxon signed rank test was used for cadence, walk ratio, stride width, and MoCA score. Pre–post differences are considered significant when *p* < 0.05.

## 3. Results

All the results are detailed in Table 2. After three months of weekly MR activity, several gait spatiotemporal parameters (STPs) had significantly increased, namely walking speed (pre = 0.78 ± 0.22 m·s^−1^ vs. post = 0.89 ± 0.30 m·s^−1^; *p* = 0.028); step length (pre = 0.45 ± 0.10 m vs. post = 0.48 ± 0.11 m; *p* = 0.048); and stride length (pre = 0.90 ± 0.21 m·s^−1^ vs. post = 0.97 ± 0.23 m; *p* = 0.039). Most of the other STP mean values had evolved in the direction of an improvement (Table 2), but with no significative difference. For instance, lower stride width and higher cadence, if significantly different, would indicate a better gait [4]. Cognitive abilities seemed to also improve with regard to the MoCA score increase (pre = 9.76 ± 6.20 points out of 30 vs. post = 11.18 ± 7.50 points out of 30; *p* = 0.011).

## 4. Discussion

After three months of weekly mixed reality, the walking speed, stride length, step length, and MoCA scores had significantly increased.

### 4.1. Walking Speed

#### 4.1.1. Initial Walking Speed

Before training, the initial walking speed was low (0.78 ± 0.22 m·s^−1^). It was, for instance, substantially lower than the one measured by Verlinden et al. [39] among subjects aged over 50 (1.18 ± 0.19 m·s^−1^), or the normative data from Hollman et al. [40] for subjects aged over 85 (0.98 ± 0.20 m·s^−1^). This confirmed that people with ID have an impaired gait, in line with the findings of Oppewal et al. [4] in which gait speed was 1.18 ± 0.23 m·s^−1^, also under the norms for healthy adults in a similar age range [41] (see Bohannon and Andrews, 2011 [42] for a review on normal walking speed).

#### 4.1.2. Increased Walking Speed after Training

Walking speed increased after 3 months of the MR weekly activity (pre = 0.78 ± 0.22 m·s^−1^ vs. post = 0.89 ± 0.30 m·s^−1^; *p* = 0.028), confirming the primary hypothesis. This could be related to an improvement in motor skills since walking speed is a valid, reliable, and sensitive indicator of functional motor performance [41,43]. The average walking speed of the sample thus passed the threshold of 0.8 m·s^−1^, below which some authors consider the risk of falls to be high, community ambulation limited, and the risk of frailty increased in older people [43].

Several elements may explain why gait velocity increased after this three-month mixed reality training. First, the subjects’ physical fitness level might have increased. Indeed, although light, this MR game remains a physical activity that requires standing up, moving around, and using the upper limb. In some subjects, it involved a moderate cardiorespiratory effort. People with ID often have low physical fitness levels [44]. Thus, future research on MR should also focus on the physical load that is induced by the MR training. Therefore, the light physical activity provided by the MR training might have been enough to improve their fitness level. Several indicators of good fitness level (i.e., greater muscle strength, flexibility, and maximum oxygen uptake) are linked to improved gait parameters such as increased stride length [45].

Second, the walking speed increase might have been due to a significant increase in the stride length (pre = 0.90 ± 0.21 m·s^−1^ vs. post = 0.97 ± 0.23 m; *p* = 0.039) and step length (pre = 0.45 ± 0.10 m vs. post = 0.48 ± 0.11 m; *p* = 0.048), since velocity is the product of cadence and stride or step length. If stride and step lengths increased under the same experimental conditions and with the same indications (walking at a comfortable self-selected speed), it may be because the subjects’ physical resources are better [46]. These stride lengths may therefore reflect better dynamic balance during walking [46]. Furthermore, the improvement in velocity was significant whereas it was not for the majority of the STPs. As walking speed is a functional result of spatiotemporal and kinematic parameters, a complementary study focusing on kinematics analysis might help identify which segmental coordinations are involved in modifying gait.

#### 4.1.3. Sensorimotor Functions Improvement

In the present study, pre- and post-walking speed mean values were close to those in Azadian et al. [8] (i.e., pre: 0.76 m/s vs. post: 0.92 m/s) in older adults. In the Azadian et al. study, the activity carried out was only cognitive, but the training load was much higher (3 × 45 min/week in Azadian et al. vs. 1 × 15–25 min/week in the present study).

Thus, this mixed reality training appeared more efficient than an only cognitive one to improve motor skills. This can be explained by the fact that this MR activity stimulated sensorimotor functions. While playing, subjects were therefore performing a perceptual-motor task. Hence, our results are in line with other studies in which multi-modal training has been shown to be efficient for improving motor skills in pathological populations such as dual-task training (i.e., cerebral palsy [47]; stroke [31]; Parkinson disease [48]) or perceptual-motor training (i.e., athletes with sports-related concussion history [49] or adults with ID and autism spectrum disorder [50]).

Impaired sensorimotor functions may affect gait pattern, especially reducing step length [51] and thus walking speed. Thus, as the present study training involved a perceptual-motor activity, the increase in walking speed and step length might be explained by an improvement in the subjects’ sensorimotor functions.

### 4.2. Cognitive Ability Improvement

Cognitive abilities as evidenced by the MoCA score improved after 3 months of weekly MR activity (pre = 9.76 ± 6.20 points out of 30 vs. post = 11.18 ± 7.50 points out of 30; *p* = 0.011), confirming the secondary hypothesis. As a note, people with ID are usually subject to a natural decline of cognitive functions [52]. However, the three-month study period was probably not long enough for this decline to be noticeable with the MoCA.

The present study results showed a significant improvement of the MoCA score, as observed by de Andrade et al. [29] (i.e., pre = 12.6 ± 5.7 vs. post = 16.0 ± 5.7; *p* = 0.01). In this study, older adults with Alzheimer’s disease performed dual-task training. However, once again, the training load was much higher (i.e., 3 × 1 h/week for 16 weeks, including 25 min for warm-up and aerobic work in the beginning of each session). In comparison, the present study’s perceptual-motor MR training therefore improved cognitive abilities despite a low training load.

The MoCA is generally used because it is quick to administer and gives a general score of cognitive ability [32]. However, it might not be sufficiently discriminating to provide an accurate and detailed picture of the subjects’ cognitive abilities. For example, several subjects improved in some exercises without the corresponding point being attributed according to the MoCA evaluation grid (e.g., for the exercise where a sequence of five digits must be repeated, several subjects increased the number of digits repeated without reaching five, which is still worth zero points).

### 4.3. Added Value of Mixed Reality

Based on the results, MR seems to be a great tool to provide effective perceptual-motor training. Further evidence of its efficiency is that another study [53], with a similar slight multi-modal training protocol, but not in MR (i.e., walking while doing an upper limb motor task, a cognitive task, or both) showed no effect on the subjects’ gait. According to the authors, the protocol application duration was insufficient.

Moreover, comparatively to other studies using extended reality [14,31], subjects thoroughly enjoyed practicing and were therefore very involved. According to Bioulac et al. [14], children with psychiatric disorders are very receptive to these kinds of technologies. This was also the case in this study despite the advanced age of some of the subjects. In addition, although riskier than computerized cognitive exercises, this MR task shows less risk [54] and physical demand than traditional physical activity. The feasibility of MR as a therapeutic tool with intellectually disabled individuals is thus validated.

### 4.4. Limits

The main limit of this study was the lack of a control group. Thus, it is difficult to state that the changes were only due to the training in MR. Nevertheless, as they were residents of the same establishment, most of their other weekly activities were common and rather constant before, during, and after the study. Furthermore, it is a preliminary study; a comparison study with a control group will be carried out in the future.

Also, the study sample showed some heterogeneity in its characteristics (i.e., age, level of ID, initial walking speed, etc.). However, the standard deviations (sd) of STPs were similar to other studies, such as walking speed (sd = 0.20 m·s^−1^ in the present study; sd = 0.23 m·s^−1^ in Verlinden et al. [39]; sd = 0.19 m·s^−1^ in Oppewal et al. [4]) and the level of ID was not associated with the gait characteristics [4]. Furthermore, it made the sample representative of the population of adults with intellectual disability.

## 5. Conclusions and Perspectives

This pilot study is the first to show that a mixed reality training can improve walking speed and cognitive abilities in people with intellectual disability, despite a low training load. These improvements might be explained by the fact that the activity involved perceptual-motor tasks (seeking out, detecting, walking, pointing, etc.). This opens the door to the use of MR with this audience, who incidentally thoroughly enjoyed the activity.

Increased walking speed in this pilot study was the first indicator of motor improvement after MR training in people with ID. However, future research should focus on other motor parameters, such as gait kinematics or motor coordination, to investigate what processes are involved in this improvement. To provide accurate recommendations for the use of MR with this population, it might be interesting to carry out case-by-case analyses, or conduct statistical classifications (e.g., PCA, cluster analysis, etc.) including the above-mentioned parameters. 

## Figures and Tables

**Table 1:** Characteristics of the study sample.

	Study Sample (18 Subjects)	
*Age*		
	years, m ± sd, range	44 ± 14, 23–69
*Women*		
	n (%)	6 (33.3%)
	Height (m), m ± sd	1.57 ± 0.10
	Mass (kg) m ± sd	60.67 ± 14.9
*Men*		
	n (%)	12 (66.7%)
	Height (m) m ± sd	1.73 ± 0.04
	Mass (kg) m ± sd	85.4 ± 31.0
*Known co-disorders*		
	ASD, n (%)	4 men (22.2%)

m = mean; sd = standard deviation; n = number; ASD = autistic spectrum disorders.

**Table 2:** Gait spatiotemporal parameters and MoCA score before and after the three-month mixed reality training.

		Pre	Post	*p*-Value (Statistic Test)
		m ± sd	m ± sd	
*Gait spatiotemporal parameters*				
Speed,	cm/s	78.13 ± 22.03	89.10 ± 29.85	0.028 *
cadence,	step/min	101.40 ± 13.59	104.55 ± 19.38	0.382
Walk Ratio,	cm/step/min	0.45 ± 0.11	0.46 ± 0.08	0.314
Step length,	cm	44.78 ± 10.33	48.12 ± 11.20	0.048 *
Stride length,	cm	89.58 ± 20.87	97.40 ± 22.77	0.039 *
Stride width,	cm	12.96 ± 4.33	12.76 ± 4.27	0.700
Step time,	s	0.59 ± 0.08	0.57 ± 0.11	0.132
Sride time,	s	1.18 ± 0.17	1.16 ± 0.23	0.215
Stance phase,	s	0.77 ± 0.14	0.75 ± 0.17	0.372
Stance phase,	%GC	65.85 ± 3.31	65.79 ± 3.26	0.472
Single support time,	%GC	33.86 ± 3.02	34.21 ± 4.22	0.777
Double support time,	%GC	31.41 ± 6.81	32.44 ± 6.52	0.306
Initial double support time,	%GC	16.30 ± 3.09	16.19 ± 3.32	0.744
Swing phase,	%GC	34.12 ± 3.28	34.21 ± 3.26	0.472
Feet angle asymetry,		6.66 ± 5.40	7.39 ± 5.79	0.602
*Montréal Cognitive Assement*				
MoCA,	score/30	9.76 ± 6.20	11.18 ± 7.50	0.011 ^†^

m = mean; sd = standard deviation; %GC = percentage of the gait cycle; * *p* < 0.050 (paired Student *t*-test); ^†^ *p* < 0.050 (Wilcoxon signed rank test).

## Data Availability

The data are not publicly available due to privacy and ethical restrictions.

## References

[1] American Psychiatric Association (2013). Diagnostic and Statistical Manual of Mental Disorders.

[2] Enkelaar L., Smulders E., van Schrojenstein Lantman-de Valk H., Geurts A.C.H., Weerdesteyn V. (2012). A review of balance and gait capacities in relation to falls in persons with intellectual disability. Res. Dev. Disabil..

[3] Almuhtaseb S., Oppewal A., Hilgenkamp T.I.M. (2014). Gait characteristics in individuals with intellectual disabilities: A literature review. Res. Dev. Disabil..

[4] Oppewal A., Festen D.A.M., Hilgenkamp T.I.M. (2018). Gait Characteristics of Adults with Intellectual Disability. Am. J. Intellect. Dev. Disabil..

[5] Carmeli E., Bar-Yossef T., Ariav C., Levy R., Liebermann D.G. (2008). Perceptual-motor coordination in persons with mild intellectual disability. Disabil. Rehabil..

[6] Smulders E., Enkelaar L., Weerdesteyn V., Geurts A.C.H., van Schrojenstein Lantman-de Valk H. (2012). Falls in older persons with intellectual disabilities: Fall rate, circumstances and consequences: Falls in persons with ID. J. Intellect. Disabil. Res..

[7] Diamond A. (2000). Close Interrelation of Motor Development and Cognitive Development and of the Cerebellum and Prefrontal Cortex. Child Dev..

[8] Azadian E., Torbati H.R.T., Kakhki A.R.S., Farahpour N. (2016). The effect of dual task and executive training on pattern of gait in older adults with balance impairment: A Randomized controlled trial. Arch. Gerontol. Geriatr..

[9] Hillman C.H., Erickson K.I., Kramer A.F. (2008). Be smart, exercise your heart: Exercise effects on brain and cognition. Nat. Rev. Neurosci..

[10] Bayot M., Dujardin K., Tard C., Defebvre L., Bonnet C.T., Allart E., Delval A. (2018). The interaction between cognition and motor control: A theoretical framework for dual-task interference effects on posture, gait initiation, gait and turning. Neurophysiol. Clin..

[11] Künstler E.C.S., Finke K., Günther A., Klingner C., Witte O., Bublak P. (2018). Motor-cognitive dual-task performance: Effects of a concurrent motor task on distinct components of visual processing capacity. Psychol. Res..

[12] Hamacher D., Herold F., Wiegel P., Hamacher D., Schega L. (2015). Brain activity during walking: A systematic review. Neurosci. Biobehav. Rev..

[13] Al-Issa H., Regenbrecht H., Hale L. (2012). Augmented reality applications in rehabilitation to improve physical outcomes. Phys. Ther. Rev..

[14] Bioulac S., de Sevin E., Sagaspe P., Claret A., Philip P., Micoulaud-Franchi J., Bouvard M. (2018). Qu’apportent les outils de réalité virtuelle en psychiatrie de l’enfant et l’adolescent ?. L’Encéphale.

[15] Kim S., Ryu J., Choi Y., Kang Y., Li H., Kim K. (2020). Eye-Contact Game Using Mixed Reality for the Treatment of Children with Attention Deficit Hyperactivity Disorder. IEEE Access.

[16] Levin M.F., Demers M. (2021). Motor learning in neurological rehabilitation. Disabil. Rehabil..

[17] Klinger E., Chemin I., Lebreton S., Marié R.-M. (2006). Virtual Action Planning in Parkinson’s Disease: A Control Study. Cyberpsychol. Behav..

[18] Prasertsakul T., Kaimuk P., Chinjenpradit W., Limroongreungrat W., Charoensuk W. (2018). The effect of virtual reality-based balance training on motor learning and postural control in healthy adults: A randomized preliminary study. Biomed. Eng. Online.

[19] Lopes J.B.P., Duarte N.d.A.C., Lazzari R.D., Oliveira C.S. (2020). Virtual reality in the rehabilitation process for individuals with cerebral palsy and Down syndrome: A systematic review. J. Bodyw. Mov. Ther..

[20] Lotan M., Weiss P.L. (2021). Improving Balance in Adults with Intellectual Developmental Disorder via Virtual Environments. Percept. Mot. Skills.

[21] Hughes C.L., Fidopiastis C., Stanney K.M., Bailey P.S., Ruiz E. (2020). The Psychometrics of Cybersickness in Augmented Reality. Front. Virtual Real..

[22] Kaufeld M., De Coninck K., Schmidt J., Hecht H. (2022). Chewing gum reduces visually induced motion sickness. Exp. Brain Res..

[23] Vovk A., Wild F., Guest W., Kuula T. Simulator Sickness in Augmented Reality Training Using the Microsoft HoloLens. Proceedings of the 2018 Conference on Human Factors in Computing Systems.

[24] Kirollos R., Merchant W. (2023). Comparing Cybersickness in Virtual Reality and Mixed Reality Head-Mounted Displays. Front. Virtual Real..

[25] Glueck A.C., Han D.Y. (2020). Improvement potentials in balance and visuo-motor reaction time after mixed reality action game play: A pilot study. Virtual Real..

[26] Laly A., Biard J.C., Tixier P.A., Ferrari A., Rosnet E., Houel N. Influence of a Mixed Reality Training on Gait in People with Mental Disabilities. Proceedings of the 28th Congress of the International Society of Biomechanics, ISB 2021.

[27] Pellecchia G.L. (2005). Dual-Task Training Reduces Impact of Cognitive Task on Postural Sway. J. Motor Behav..

[28] Li K.Z.H., Roudaia E., Lussier M., Bherer L., Leroux A., McKinley P.A. (2010). Benefits of Cognitive Dual-Task Training on Balance Performance in Healthy Older Adults. J. Gerontol. A Biol. Sci. Med. Sci..

[29] de Andrade L.P., Gobbi L.T.B., Coelho F.G.M., Christofoletti G., Costa J.L.R., Stella F. (2013). Benefits of Multimodal Exercise Intervention for Postural Control and Frontal Cognitive Functions in Individuals with Alzheimer’s Disease: A Controlled Trial. J. Am. Geriatr. Soc..

[30] Atak E., Algun Z.C. (2022). Dual-Task Balance Training for Motor Skill Development among Children with Intelligence Quotient Discrepancy. Rehabil. Res. Pract..

[31] Fishbein P., Hutzler Y., Ratmansky M., Treger I., Dunsky A. (2019). A Preliminary Study of Dual-Task Training Using Virtual Reality: Influence on Walking and Balance in Chronic Poststroke Survivors. J. Stroke Cerebrovasc. Dis..

[32] Nasreddine Z.S., Phillips N.A., Bédirian V., Charbonneau S., Whitehead V., Collin I., Cummings J.L., Chertkow H. (2005). The Montreal Cognitive Assessment, MoCA: A Brief Screening Tool for Mild Cognitive Impairment. J. Am. Geriatr. Soc..

[33] Smith T., Gildeh N., Holmes C. (2007). The Montreal Cognitive Assessment: Validity and Utility in a Memory Clinic Setting. Can. J. Psychiatry.

[34] Pelletier S., Nalpas B., Alarcon R., Rigole H., Perney P. (2016). Investigation of Cognitive Improvement in Alcohol-Dependent Inpatients Using the Montreal Cognitive Assessment (MoCA) Score. J. Addict..

[35] Bernstein I.H., Lacritz L., Barlow C.E., Weiner M.F., DeFina L.F. (2011). Psychometric Evaluation of the Montreal Cognitive Assessment (MoCA) in Three Diverse Samples. Clin. Neuropsychol..

[36] Vallabhajosula S., Humphrey S.K., Cook A.J., Freund J.E. (2019). Concurrent Validity of the Zeno Walkway for Measuring Spatiotemporal Gait Parameters in Older Adults. J. Geriatr. Phys. Ther..

[37] Lynall R.C., Zukowski L.A., Plummer P., Mihalik J.P. (2017). Reliability and validity of the protokinetics movement analysis software in measuring center of pressure during walking. Gait Posture.

[38] Inman V.T., Ralston H.J., Todd F. (1981). Human Walking.

[39] Verlinden V., van der Geest J., Hoogendam Y., Hofman A., Breteler M., Ikram M. (2013). Gait patterns in a community-dwelling population aged 50 years and older. Gait Posture.

[40] Hollman J.H., McDade E.M., Petersen R.C. (2011). Normative spatiotemporal gait parameters in older adults. Gait Posture.

[41] Middleton A., Fritz S.L., Lusardi M. (2015). Walking Speed: The Functional Vital Sign. J. Aging Phys. Act..

[42] Bohannon R.W., Williams Andrews A. (2011). Normal walking speed: A descriptive meta-analysis. Physiotherapy.

[43] Graham J.E., Ostir G.V., Fisher S.R., Ottenbacher K.J. (2008). Assessing walking speed in clinical research: A systematic review. J. Eval. Clin. Pract..

[44] Hilgenkamp T.I.M., van Wijck R., Evenhuis H.M. (2012). Low physical fitness levels in older adults with ID: Results of the HA-ID study. Res. Dev. Disabil..

[45] Choi H., Lim J., Lee S. (2021). Body Fat-Related Differences in Gait Parameters and Physical Fitness Level in Weight-Matched Male Adults. Clin. Biomech..

[46] Farrell J.W., Merkas J., Pilutti L.A. (2020). The Effect of Exercise Training on Gait, Balance, and Physical Fitness Asymmetries in Persons with Chronic Neurological Conditions: A Systematic Review of Randomized Controlled Trials. Front. Physiol..

[47] Okur E.O., Arik M.I., Okur I., Gokpinar H.H., Gunel M.K. (2022). Dual-task training effect on gait parameters in children with spastic diplegic cerebral palsy: Preliminary results of a self-controlled study. Gait Posture.

[48] Yogev-Seligmann G., Giladi N., Brozgol M., Hausdorff J.M. (2012). A Training Program to Improve Gait While Dual Tasking in Patients with Parkinson’s Disease: A Pilot Study. Arch. Phys. Med. Rehabil..

[49] Wilkerson G.B., Nabhan D.C., Crane R.T. (2021). Upper-Extremity Perceptual-Motor Training Improves Whole-Body Reactive Agility Among Elite Athletes with History of Sport-Related Concussion. J. Sport Rehabil..

[50] Azar N.R., McKeen P., Carr K., Sutherland C.A., Horton S. (2016). Impact of motor skills training in adults with autism spectrum disorder and an intellectual disability. J. Dev. Disabil..

[51] Menz H.B., Lord S.R., Fitzpatrick R.C. (2007). A structural equation model relating impaired sensorimotor function, fear of falling and gait patterns in older people. Gait Posture.

[52] Krinsky Mchale S., Silverman W. (2013). Dementia and mild cognitive impairment in adults with intellectual disability: Issues of diagnosis. Dev. Disabil. Res. Rev..

[53] Souza F.H.N., Lisboa A.K.P., Maia M.D.F.T., Dos Santos M.R.D., Silva Filho E.M., Cezarino L.G., Cacho R.O., Cacho Ê.W.A. (2017). Effects of dual task training on gait temporal-spatial parameters of children with autism. Man. Ther. Posturol. Rehabil. J..

[54] Barteit S., Lanfermann L., Bärnighausen T., Neuhann F., Beiersmann C. (2021). Augmented, Mixed, and Virtual Reality-Based Head-Mounted Devices for Medical Education: Systematic Review. JMIR Serious Games.

